# You Are What You Eat: Within-Subject Increases in Fruit and Vegetable Consumption Confer Beneficial Skin-Color Changes

**DOI:** 10.1371/journal.pone.0032988

**Published:** 2012-03-07

**Authors:** Ross D. Whitehead, Daniel Re, Dengke Xiao, Gozde Ozakinci, David I. Perrett

**Affiliations:** 1 School of Psychology, Perception Lab, University of St Andrews, St Andrews, Fife, Scotland; 2 School of Medicine, University of St Andrews, St Andrews, Fife, Scotland; University of Illinois at Champaign-Urbana, United States of America

## Abstract

**Background:**

Fruit and vegetable consumption and ingestion of carotenoids have been found to be associated with human skin-color (yellowness) in a recent cross-sectional study. This carotenoid-based coloration contributes beneficially to the appearance of health in humans and is held to be a sexually selected cue of condition in other species.

**Methodology and Principal Findings:**

Here we investigate the effects of fruit and vegetable consumption on skin-color longitudinally to determine the magnitude and duration of diet change required to change skin-color perceptibly. Diet and skin-color were recorded at baseline and after three and six weeks, in a group of 35 individuals who were without makeup, self-tanning agents and/or recent intensive UV exposure. Six-week changes in fruit and vegetable consumption were significantly correlated with changes in skin redness and yellowness over this period, and diet-linked skin reflectance changes were significantly associated with the spectral absorption of carotenoids and not melanin. We also used psychophysical methods to investigate the minimum color change required to confer perceptibly healthier and more attractive skin-coloration. Modest dietary changes are required to enhance apparent health (2.91 portions per day) and attractiveness (3.30 portions).

**Conclusions:**

Increased fruit and vegetable consumption confers measurable and perceptibly beneficial effects on Caucasian skin appearance within six weeks. This effect could potentially be used as a motivational tool in dietary intervention.

## Introduction

Carotenoids are yellow-red organic pigments which are abundant in, and impart color to, fruit and vegetables. These phytochemicals are efficient singlet oxygen quenchers [1] which enables them to protect tissue against oxidative stress, arising when the balance of oxidants to antioxidants *in vivo* is in favor of the former. Such conditions can precipitate damage to cellular proteins, lipids and DNA [1] and consequently may contribute to a variety of age-related degenerative processes [2], cardiovascular disease [3], diabetes and related complications [4], [5] and possibly some cancers [6].

In addition to the endogenous oxidants produced as part of normal metabolic [7] and immunological processes [8], skin is directly exposed to a number of environmental oxidants, such as UV radiation, nitrogen oxides, cigarette smoke and ozone [9]. As antioxidants, carotenoids are important for skin health, serving a protective role by virtue of their relatively high concentration in all layers of this organ [10]–[13]. Illustrating their importance in this capacity, carotenoids reduce ultraviolet light (UV) sensitivity, increasing the minimum level of UV exposure required to induce erythema [12], [14], [15]. Carotenoids are expended in their antioxidative role [16], potentially leading to organism-wide deficits which, if not restored via dietary intake, could precipitate conditions associated with elevated oxidative stress [2]–[6]. Further, carotenoids are implicated in immune-cell activity [17], [18], increasing the cell-surface expression of MHC class II signaling molecules [19], [20] - consequently, carotenoid deficits may also lead to immune suppression.

The accumulation of carotenoids causes these fat-soluble pigments to impart color to human skin, in particular, contributing to normal skin yellowness [12], [21]. Carotenoids may accrue in the skin via two mechanisms, one of which involves carotenoids being deposited onto, and assimilated into, the lipophilic stratum corneum via sebaceous and eccrine activity [11], [22]. A second process, which may have a greater influence on short-term fluctuations in skin carotenoid levels, involves diffusion of carotenoids from the skin's rich capillary network to the dermis and epidermis [22].

Individual differences in dietary intake of carotenoids, which occur chiefly via fruit and vegetable consumption, have been linked to between-subject variation in skin yellowness [23], in particular the b* axis of the CIE 1976 L*a*b* color-opponent space (where L* represents lightness and positive values of a* and b* represent degrees of redness and yellowness, respectively). Studies using Raman spectroscopy [22], [24] indicate that skin carotenoid concentrations are also subject to short-term fluctuation within individuals. This variation was linked to diet change, carotenoid supplementation and other important lifestyle factors such as tobacco use, alcohol consumption and infectious illness which are known to affect oxidant and antioxidant levels [8], [25]–[28].

Variation in carotenoid-based coloration affects the appearance of healthiness. When participants are able to digitally manipulate the skin-color of calibrated Caucasian [23], African [23] and Asian face stimuli [Whitehead et al., unpublished data], yellowness (b*) is consistently increased for both own ethnicity and other ethnicity faces. A large body of research on bird and fish coloration also suggests that carotenoid-based pigmentation is an indicator of healthy condition [29]. In this literature, carotenoid coloration is widely held to be an honest indicator of quality due to a necessary trade-off between carotenoid expenditure as antioxidants and display in ornamentation [30]. This trade-off reflects the organism's prevailing oxidative stress and hence overall condition. Variation in oxidative stress can be caused by either increased exposure to pro-oxidants and/or via a depleted antioxidant network. The individual's health status, and hence value as a mate [31], can affect both of these factors. During phagocytosis, neutrophils create an abundance of reactive oxygen species (ROS) as part of a primary immune response to pathogens [8] necessitating increased expenditure of antioxidants, including carotenoids, to prevent cellular damage during periods of infection [32]. Furthermore, variation in the robustness of the entire antioxidant network can arise due to environmental factors such as diet [33] and endogenous factors such as enzymic antioxidant capacity, which may be increased by regular aerobic exercise [34], [35]. It follows that carotenoid display in scales, feathers or skin is most affordable in those individuals with the lowest pathogen load and in those best able to forage for [36] or maintain an adequate antioxidant buffer [29]. Preferences for carotenoid coloration are hypothesized to arise through intersexual selection [37], as more colorful mates would offer direct [38], [39] and indirect [40] fitness benefits to the choosing individual.

There are important ramifications of this research for human health. Adults in the UK and US predominantly consume fewer than 400 g of fruit and vegetables per day [41], [42]. Such inadequate intake is estimated to precipitate 2.6 million premature deaths per year worldwide [43]. The literature reviewed here suggests potential utility in forming novel dietary intervention strategies. Fruit and vegetable consumption affects skin carotenoid levels [13], [22]; this may lead to skin-color change in a fashion that is known to contribute to the appearance of health [23]. It follows that dietary change may be motivated by illustrating to individuals these beneficial effects on appearance. Despite qualitative evidence that skin carotenoid levels can be affected by diet [22], it is not known whether dietary variation is sufficient to confer visible skin-color changes within-subject; the quantitative level of diet change and timescale required to achieve skin-color change also remain obscure.

Here we address these issues using reflectance spectrophotometry to measure skin-color (CIE L*a*b*) and spectral reflectance at three time points over a six-week period. Diet was self-reported at each session via food frequency questionnaire to estimate daily fruit and vegetable consumption. We hypothesize that changes in fruit and vegetable consumption will correlate positively with changes in skin yellowness over this six-week study, and that diet-linked skin reflectance changes over this period will parallel the absorption spectra of common carotenoids.

We also present a psychophysical study which aims to estimate the level of diet change associated with perceptibly healthier or more attractive skin-coloration. We used a two-alternative forced-choice staircase design to determine color thresholds. Participants were asked to select either the healthier or more attractive of two sequentially presented face stimuli, which differed in color according to an empirically derived fruit and vegetable color axis. In accordance with previous studies [23], [44], we predict that participants will choose to increase coloration associated with carotenoid pigmentation.

## Experiment 1

### Methods

#### Ethics Statement

All procedures obtained ethical approval from the University of St Andrews Teaching and Research Ethics Committee, and prior informed written consent was obtained from all participants. All individuals were reimbursed financially for their participation at the rate of £5 per hour.

### Participants

Sixty-three undergraduate students at the University of St Andrews each participated in three sessions between March and June 2010. Twenty-five participants reporting recent sunbathing and/or use of self-tanning products, solariums and/or facial make-up (e.g., blusher/foundation) at any session were eliminated from data analyses. Three additional participants with initial overall skin lightness (see procedure) outside of two standard deviations from the mean were also excluded, leaving 35 participants in the final analyses (21 females, 14 males, mean age = 20.74, age range: 18–25, 34 (97.1%) Caucasian, 1 (2.9%) East Asian). These participants reported consuming an average of 3.41 (*SE*±0.32, min = 0.94, max = 7.80) fruit and vegetable portions per day over three sessions. Overall skin lightness, redness and yellowness were normally distributed in this sample (Kolmogorov-Smirnov tests all p≥.134).

### Procedure

Participants attended an initial measurement session and returned for two follow-up sessions after approximately three and six weeks (1^st^ interval mean days (± SE) = 20.8±0.81; 2^nd^ interval = 20.01±0.63), in which all measurements and questions were repeated. Participants completed a food frequency questionnaire [45] to establish daily fruit and vegetable intake. The questionnaire contained 63 items, 10 of which pertained to fruit and vegetable items, excluding potatoes. At each session participants were asked “How often do you currently eat each of the following food and drink items?” (2 or more times a day, once a day, 3–5 times a week, 1–2 times a week, 1–3 times a month, rarely/never). Daily consumption of these items was estimated and summed to achieve an estimate of daily fruit and vegetable intake. Participants were able to supplement the questionnaire with up to four additionally consumed items. Fruits or vegetables among these added items were included in the daily total.

Skin-color and reflectance were recorded using a Konica Minolta CM-2600d spectrophotometer. An 8 mm diameter aperture was used for all measurements. CIE L*a*b* [46] tristimulus values (where L* represents lightness and positive values of a* and b* represent degrees of redness and yellowness, respectively) and spectral reflectance at wavelengths between 400 and 540 nm at 10 nm intervals were recorded (both excluding specular reflection) for each participant at seven body locations (left cheek, right cheek, forehead, volar forearm, outer bicep, shoulder and palmar thenar eminence). Care was taken to ensure that the aperture was lightly held against skin, in order to minimize pressure-induced blanching. White-point calibration was conducted before each recording session according to a white reference tile.

### Results

Mean initial skin-color values and six-week changes are presented in Table 1. To investigate the effect of diet on skin-color, we examined the impact of changes in fruit and vegetable consumption within-subjects using linear regression, with skin-color change over each three-week interval or the entire six-week study as the dependent variable, and change in fruit and vegetable consumption over these periods as the independent variable.

**Table 1:** Mean initial CIE L*a*b* values across 35 participants.

	Mean initial value *(± SE)*	Minimum	Maximum	Mean *Δ(± SE)*	Minimum *Δ*	Maximum *Δ*
Overall L*	66.95±0.31	62.83	69.64	−0.07±0.18	−3.08	1.92
Overall a*	9.24±0.24	5.97	12.51	+0.20±0.12	−1.29	2.04
Overall b*	14.50±0.25	10.68	17.52	+0.51±0.13	−1.16	2.65
Face L*	65.94±0.39	59.41	69.53	−0.40±0.23	−3.45	1.93
Face a*	11.48±0.33	7.42	16.98	+0.30±0.21	−2.09	2.39
Face b*	14.66±0.28	11.09	18.02	+0.74±0.17	−1.43	2.54

L* represents skin lightness (0–100), a* represents position on a green-red axis (−60 to +60) where positive values are red and b* represents position on a blue-yellow axis (−60 to +60) where positive values are yellow. Overall values represent average skin-color across seven body regions (left cheek, right cheek, forehead, volar forearm, outer bicep, shoulder and palmar thenar eminence). Face values represent the average of the three facial measurements. Delta (*Δ*) values represent six-week changes in skin-color values.

Spearman correlation was used to investigate the link between diet change and skin reflectance change at wavelengths between 400 nm and 540 nm. We then investigated, using further Spearman correlations, whether the strength of this relationship followed the absorption spectra of common carotenoids [47] or melanin [48], which also affects skin yellowness [49].

**Figure 1:**
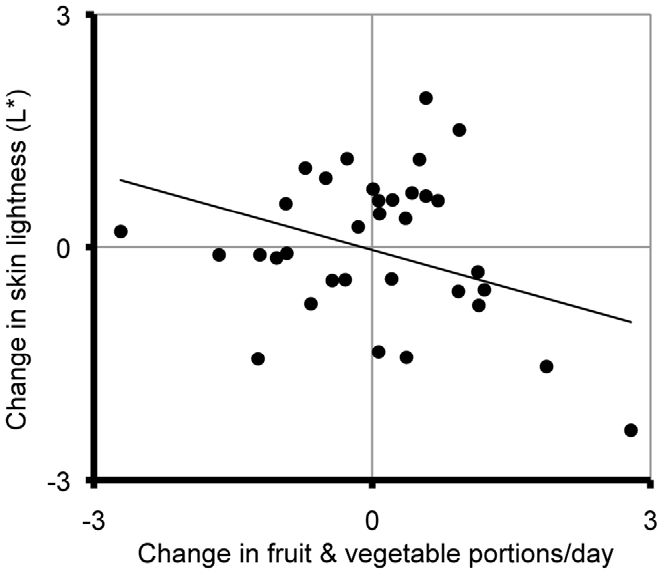
The relationship between six-week changes in fruit and vegetable intake and average skin lightness (L*).

**Figure 2:**
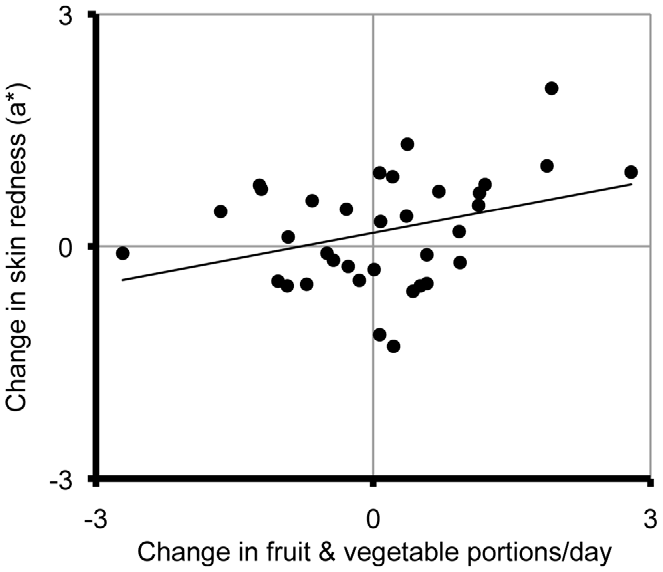
The relationship between six-week changes in fruit and vegetable intake and average skin redness (a*).

**Figure 3:**
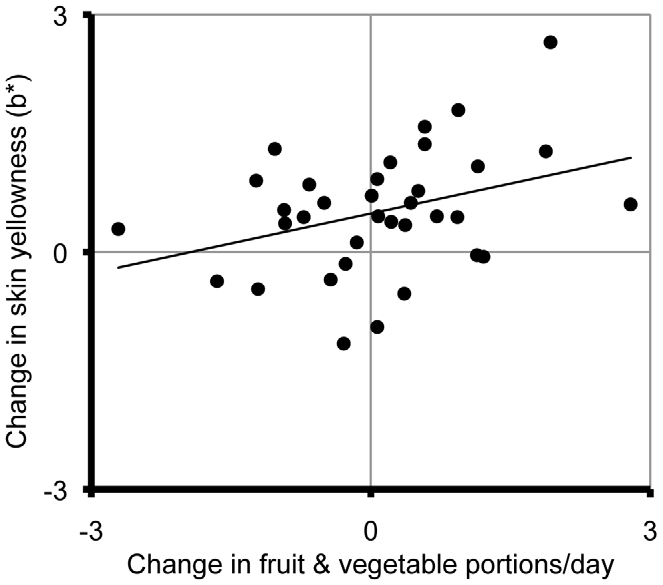
The relationship between six-week changes in fruit and vegetable intake and average skin yellowness (b*).

**Figure 4:**
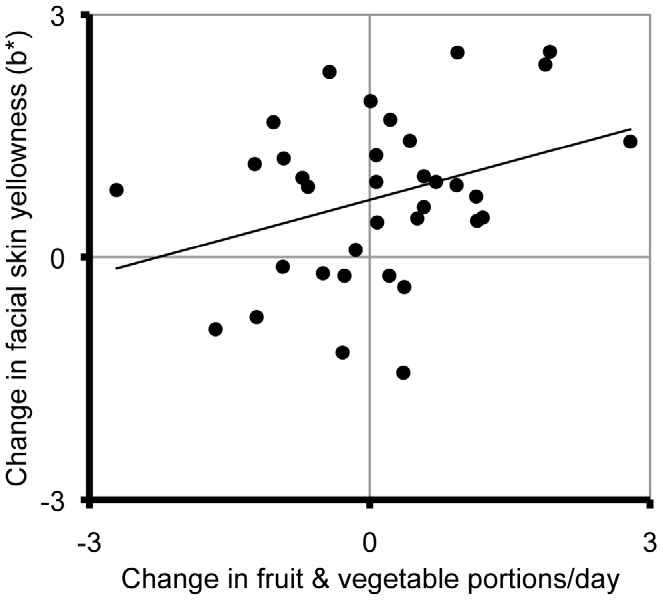
The relationship between six-week changes in fruit and vegetable intake and average facial skin yellowness (b*).

Over the six-week study period, skin lightness decrease averaged across all seven measured regions was significantly associated with increase in fruit and vegetable consumption (*b* = −0.333, SE*_b_* = 0.163, p = .049, *r* ^2^ = .11; Figure 1). Skin redness (*b* = 0.224, SE*_b_* = 0.108, p = .045, *r* ^2^ = .12; Figure 2) and yellowness (*b* = 0.251, SE*_b_* = 0.116, p = .038, *r* ^2^ = .12; Figure 3) changes were significantly associated with increase in fruit and vegetable intake. Averaged across the three facial measurements, neither skin lightness (*b* = −0.299, SE*_b_* = 0.212, p = .169, *r* ^2^ = .06) nor redness (*b* = 0.151, SE*_b_* = 0.200, p = .456, *r* ^2^ = .02) changes were significantly associated with change in fruit and vegetable consumption. Increase in fruit and vegetable intake over the six-week period was marginally associated with increases in facial skin yellowness (*b* = 0.312, SE*_b_* = 0.154, p = .051, *r* ^2^ = .11; Figure 4). Examination of changes over the three-week periods between participants' first two, or last two sessions revealed no significant relationships between fruit and vegetable intake change and skin-color change (all *p*≥.229).

To investigate whether the observed relationships between diet change and skin-color change were contingent on baseline diet a series of ANCOVA models were constructed, each with skin-color change over the six-week period as the dependent variable and change in fruit and vegetable consumption over this period and initial fruit and vegetable consumption as covariates. These models revealed no impact of initial fruit and vegetable consumption on overall or facial skin lightness, redness or yellowness change (all *F*≤2.321, *p*≥.137, *η_p_^2^*≤0.07). The effect of six-week diet change on change in skin-color remained as above, i.e. diet change was significantly associated with change in overall lightness, redness and yellowness (all *F*≥4.181, *p*≤0.049, *η_p_^2^*≥0.116) and marginally significantly associated with change in facial yellowness (*F* = 3.974, *p* = .055, *η_p_^2^* = 0.11). Over the six-week period, the skin-color change (*ΔE)* associated with an increase of one portion of fruit and vegetables per day was 0.47 over all seven body measurements and 0.46 for facial skin (*ΔE* is a standard means of presenting color difference in CIE space, and here represents the Euclidean distance between zero and the unstandardized beta values for L*, a* and b* change derived in the linear regression analyses).

To investigate further the basis of the observed skin-color changes over this six-week period we examined changes in skin reflectance at 400–540 nm, the wavelengths associated with peak light absorption by carotenoids [47]. To control for the impact of between-subject differences in overall skin reflectance, recorded values were normalized by dividing each raw reflectance value by average reflectance across all measured wavelengths [23]. Following a modified version of the methods used in Stephen et al [23], at 10 nm intervals, we obtained Spearman's rank correlation coefficients for the relationships between change in skin reflectance and change in fruit and vegetable consumption over the six-week period of the study (ρ *_Δ reflectance vs Δ diet_)*. Negative correlations are expected because, if an increase in the consumption of carotenoid-rich fruit and vegetables leads to the deposition of these pigments in the skin, then the skin's absorption will increase at these wavelengths, producing a concurrent decrease in reflectance.

**Figure 5:**
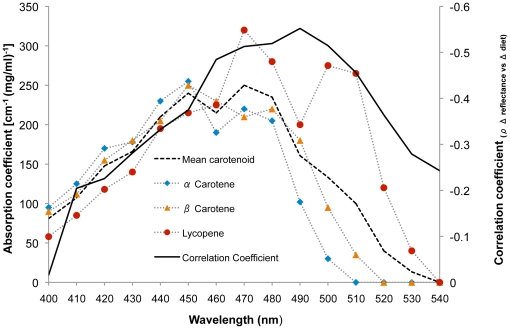
Diet-linked skin reflectance changes track common carotenoids. Solid black line - Spearman correlation coefficients (_ρ *Δ* reflectance vs *Δ* diet_) of the relationships between fruit and vegetable consumption change (from week 0 to 6) and average skin reflectance change. Dashed bold line shows the mean absorption spectra [cm^−1^ (mg/ml)^−1^] for three common carotenoids, which are individually plotted (blue rhombi - α-carotene, orange triangles - β-carotene and red circles - lycopene).

Spearman correlation was then used to examine whether the strength of this relationship (ρ *_Δ reflectance vs Δ diet_*) is associated with carotenoid absorption across the 400–540 nm wavelength range [47]. We expect the relationship to be strongest (more negative) at wavelengths associated with the greatest light absorption by carotenoids, and weaker at wavelengths associated with lower absorption. Across wavelengths 400–540 nm, the relationship between overall skin reflectance change (average of all seven measured skin regions) and fruit and vegetable consumption change from week zero to six was not significantly correlated with the absorption spectra of α-carotene (ρ = −0.232, *p* = .404) but was marginally significantly correlated with the absorption spectra of β-carotene (ρ = −0.454, *p* = .089) and significantly correlated with the absorption spectra of lycopene (ρ = −0.846, *p*<.001) and the mean absorption across these three common carotenoids (ρ = −0.539, *p* = .038; Figure 5).

**Figure 6:**
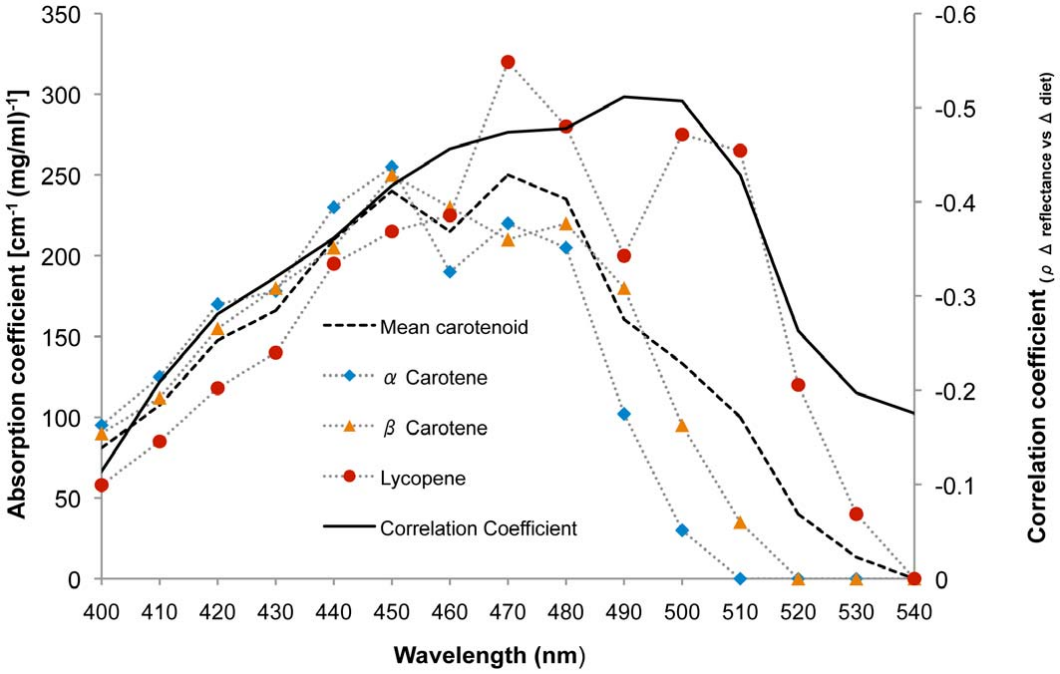
Diet-linked facial skin reflectance changes track common carotenoids. Solid black line - Spearman correlation coefficients (_ρ *Δ* reflectance vs *Δ* diet_) of the relationships between fruit and vegetable consumption change (from week 0 to 6) and facial skin reflectance change. Dashed bold line shows the mean absorption spectra [cm^−1^ (mg/ml)^−1^] for three common carotenoids, which are individually plotted (blue rhombi - α-carotene, orange triangles - β-carotene and red circles - lycopene).

Over the six-week duration, the strength of relationship between facial (average of three face regions) skin reflectance change and fruit and vegetable consumption change was not significantly correlated with the absorption spectra of α-carotene (*ρ* = −0.386, *p* = .156), but was significantly correlated with the absorption spectra of β-carotene, lycopene and the mean absorption of these three carotenoids (all *ρ*<−0.588, *p*≤.021; Figure 6). The relationship between average skin, or facial skin reflectance change and fruit and vegetable consumption change was not significantly correlated with the absorption spectrum of melanin [48] over these wavelengths (both *ρ*≤0.399, *p*≥.140).

The results remain similar when we repeated the above analyses controlling for the potential effect of initial fruit and vegetable consumption. To achieve this we conducted Spearman correlations between skin reflectance change and standardized residuals derived from a non-significant linear regression with six-week diet change as the dependent variable and initial diet as the independent variable (*b* = −0.004, SE*_b_* = 0.103, *p* = .970, *r* ^2^ = .00). This partials out any effect of starting diet on diet change. Over all measured body regions, the strength of these correlations was not significantly associated with the absorption spectra of α-carotene (*ρ* = −0.205, *p* = .463) or β-carotene (*ρ* = −0.427, *p* = .113), but was significantly associated with the absorption spectra of lycopene (*ρ* = −0.829, *p*<.001) and marginally correlated with the mean absorption spectra of these three carotenoid pigments (*ρ* = −0.511, *p* = .052). For average facial skin reflectance, no association was seen with the absorption spectra of α-carotene (*ρ* = −0.386, *p* = .156) but the correlation strength was associated with the absorption spectra of β-carotene (*ρ* = −0.588, *p* = .021), lycopene (ρ = −0.893, p<.001) and the mean absorption spectra of these three carotenoids (*ρ* = −0.668, *p* = .007). No association with the absorption spectra of melanin was seen for overall (*ρ* = 0.435, *p* = .105) or facial skin (*ρ* = 0.138, *p* = .623).

### Discussion

The results of Experiment 1 reveal that fruit and vegetable consumption changes over a six-week period are sufficient to confer measurable skin-color changes over this interval. No effect of initial diet was seen on observed skin-color changes. Diet-linked skin reflectance changes were associated with the absorption spectra of common carotenoids, but not that of melanin and this pattern of results was similar once controlling for initial diet. Experiment 2 investigates the level of skin-color change required to improve apparent healthiness and attractiveness.

## Experiment 2

### Methods

#### Participants

Twenty-four undergraduate students (19 females, five males, mean age = 18.88, age range: 18–22) at the University of St Andrews participated in an investigation of the effect of skin-color changes on the perception of health and attractiveness.

### Procedure

Psychophysical methods were used to determine the minimum color change associated with fruit and vegetable consumption necessary to change viewers' perception of facial skin-color, health and attractiveness.

Images of four Caucasian individuals (two females, two males, age range: 19–21) were taken using a Fujifilm FinePix S5Pro digital SLR camera (60 mm fixed length lens) in a booth painted with achromatic matt grey paint. Illumination was exclusively provided by three 6504 K bulbs (VeriVide, Ltd). The camera was white-balanced according to a GregtagMacbeth white balance card in these lighting conditions. Participants held a grey painted board over their shoulders to occlude reflections from clothing. A GregtagMacbeth Mini ColorChecker was included in each image to color-calibrate images. Images were color-corrected by transforming observed values of each of the 24 color-checker patches towards known values of these same patches using a least-squares transform from an 11-expression polynomial expansion [23], [44], [50], [51].This resulted in a mean color error (*ΔE*) of 2.38 (*SD*±0.06), *ΔE* here represents the color difference between the calibrated image and known ColorChecker patch values.

Two face-shaped uniform color masks were created in Matlab [23], [44], [51], to represent the average skin-color of 15 high and 15 low Caucasian fruit and vegetable consumers, according to empirically derived values obtained in a previous cross-sectional experiment (Table 2; Whitehead et al., unpublished data). The two groups were equivalent in terms of gender (five males, 10 females in each group), age [low = 20.75±1.67 (*M* ± *SD*), high = 20.99±2.29], hours of vigorous exercise per week (high = 0.87±0.52, low = 0.80±0.94) and body mass index (high = 22.0±2.65, low = 22.55±3.43, Mann-Whitney *U* test, all *p*≥.475). Individuals were without self-tanning agents, make-up and recent intensive sun exposure. In terms of per-portion difference, the color-values between the two groups are very similar to those obtained for diet change in Experiment 1.

**Table 2:** Mean volar forearm color in CIE L*a*b* tristimulus color space (± SE).

	L*	a*	b*	Intake (F&V portions/day)
**Low fruit and vegetable consumption group**	69.56±0.62	5.28±0.39	13.18±0.56	3.08±0.19
**High fruit and vegetable consumption group**	68.56±0.48	6.42±0.19	15.30±0.37	8.63±0.56
***Δ*** ** (High-low)**	−1.00	1.14*	2.12**	5.55**

* p<.05,

** p<.005.

The skin portions (including lips and ears, excluding eyes, hair and background) of calibrated facial photographs were then manipulated according to the color difference between the two endpoint masks [52] in order to obtain a set of 22 images for each face, the middle of which was the original unmanipulated face. The masks applied were Gaussian blurred at the edges of the face (*SD* ±3 pixels), to prevent final images having an obvious color border. This continuum represented a total range of +/−1.00 L* units, +/−1.14 a* units and +/−2.12 b* units, equivalent to a change of +/−5.55 fruit and vegetable portions per day. Each of these continua reflected the color changes between high and low fruit and vegetable consumers, and within these, skin lightness, redness and yellowness changed simultaneously.

These continua were then used in a two-alternative forced-choice psychophysics computer program [53]. Face images were presented on a Sony GDM-F500R cathode ray tube monitor, color-calibrated to a *Δ* u′v′ (representing the Euclidean difference in the CIE u′v′ color space) of less than 0.5 using Spyder S2 Pro software and hardware (Pantone, Inc.). Sessions were conducted within a darkened booth, to minimize the effects of light falling on the monitor. The program sequentially showed two versions of the same face, each displayed for 750 ms, with a random dot mask presented for 100 ms in-between. Participants were asked in three separate tasks to choose the face that appeared more yellow, healthier or more attractive. Each task was tested in a separate block in random order. A staircase design [53] was used. Participants were initially shown two versions of the same face at the extreme ends of the fruit and vegetable color spectrum. The color difference was halved if the participant chose the face associated with greater fruit and vegetable consumption. A ‘reversal’ occurred when a participant chose a face color associated with reduced fruit and vegetable consumption, after which the program doubled the color difference of the previous trial. A further ‘reversal’ occurred once participants chose the face associated with greater fruit and vegetable consumption, again halving the color difference of the previous trial. Discrimination thresholds were defined as the average color difference after three reversals.

### Results

Discrimination thresholds for each of the four faces were examined across all three blocks (skin yellowness, health, and attractiveness). Repeated-measures ANOVAs found no differences in thresholds across the four faces in the tasks for skin yellowness (*F*(3) = 0.34, *p* = .80), health (*F*(3) = 1.43, *p* = .24), or attractiveness judgements (*F*(3) = 2.10, *p* = .11). We therefore collapsed thresholds across the four face stimuli when analysing thresholds across tasks. When asked to determine the yellower face, the average discrimination threshold was *ΔE* 0.89±0.08 (± *SE*), equivalent to a between-subjects change of 1.89±0.17 fruit and vegetable portions. For healthiness and attractiveness, the average thresholds were *ΔE* 1.37±0.15 and *ΔE* 1.55±0.15, equivalent to changes of 2.91±0.31 and 3.30±0.31 portions per day, respectively.

The yellowness discrimination threshold was significantly lower than the health (*t*(23) = −3.44, p = .002) and attractiveness discrimination thresholds (*t*(23) = −3.67, *p* = .001) which were not different (*t*(23) = −0.76, *p* = .45).

### Discussion

The results of Experiment 2 showed that the skin-color changes associated with fruit and vegetable consumption is seen as healthy and attractive, and is detectable at a relatively modest level of dietary change.

## Discussion

We find here that self-reported changes in diet correlate with objectively measured changes in skin-color. In addition to a positive correlation between fruit and vegetable intake changes and skin yellowness changes, we find that when all measured skin areas are combined, an increase in fruit and vegetable consumption correlates with an increase in skin redness. Such coloration is held to contribute beneficially to the appearance of health in human faces [44], [51] as is the case with skin yellowness [23], [44].

The spectral reflectance analysis conducted here indicates that the observed diet-linked changes in skin-color are attributable to the impact of carotenoids, as the relationship between diet change and skin reflectance change is strongest at wavelengths associated with light absorption by these pigments. The observed relationship between diet change and skin yellowness change is not attributable to sun exposure as we found that the diet-linked changes in skin reflectance were not associated with the absorption spectra of melanin [48].

We were unable to investigate the potential influence of constitutive pigmentation on the relationship between diet change and skin-color change as our sample was almost exclusively Caucasian and thus was limited in initial skin lightness variation. We expect our reported effects will hold in a more heterogeneous sample across the spectrum of human skin pigmentation because perceptual studies indicate that yellow (carotenoid) skin-coloration is perceived as healthy cross-culturally ([23], [Whitehead et al., unpublished data]). Nonetheless it may be the case that the magnitude of diet change required to achieve perceptible skin-color change is contingent upon initial skin lightness (i.e. the change may be less evident in dark skin). Therefore it is important to establish with further research the nature of dietary effects in non-Caucasians.

The observed relationship between overall skin redness and diet changes may reflect several processes that are not mutually exclusive. The skin's redness may be affected by fruit and vegetable consumption due to the influence of pigments such as lycopene, a red carotenoid that imparts coloration to fruits and vegetables, for example tomatoes and red peppers. This carotenoid is common in human skin [54] and may contribute to skin redness via the same mechanism by which other carotenoids such as β-carotene impact skin yellowness. In support of this view, we find that the relationship between diet changes and skin reflectance changes shows the strongest association with the absorption spectra of lycopene (Figure 4).

The relationship between diet and skin redness changes also potentially reflects the influence of fruit and vegetable consumption on the skin's blood perfusion. Polyphenols, contained in abundance in fruit and vegetables may contribute beneficially to artery elasticity and endothelium health [55], properties that may positively affect blood perfusion in the skin [56].

In the present study we found no effect of initial diet on the observed relationship between diet change and skin-color change. It is important for further work to establish the generality of these results. We might expect that individuals starting with an extremely high fruit and vegetable consumption will exhibit weaker skin-color change than those starting with a lower fruit and vegetable consumption as there may be a point at which the skin becomes saturated with dietary carotenoids. We could not find any evidence for this hypothesis within our sample but further work with a more heterogeneous sample is necessary. As population estimates of fruit and vegetable consumption [41], [42] are comparable to the range of our sample's initial self-reported intake, we hold that our results are applicable to the majority of individuals.

Studies utilizing Raman spectroscopy reveal that skin carotenoid concentrations can vary over a relatively short timescale. For example, Meinke et al [24] revealed that a carotenoid-rich dietary supplement significantly increased palm and forehead skin carotenoid concentrations within 14 days, with further accumulation when supplementation was continued for a total of 28 days. Darvin et al [22] showed carotenoid concentrations to fluctuate in palmar skin over a one to three-day period. We have found that diet and skin-color changes were only significantly correlated over a six-week interval, and not over a three week period. It may be the case that carotenoid-based changes in outwardly visible skin-color occur only via the longer-term accumulation of carotenoid pigments in the most superficial skin layers. Diet-linked decreases in skin-color may also be relatively slow, and linked to the loss of superficial skin layers via abrasion, a process that may take up to three weeks [57]. Further, subcutaneous adipose tissue accumulates lipophilic carotenoids [58] and may act as a buffer, releasing these pigments to blood and subsequently the dermis when skin concentrations reduce. Hence skin carotenoid levels may diminish only once adipose tissue is depleted of carotenoids. The mechanism responsible for more rapid fluctuation in skin carotenoid levels [22] (diffusion of carotenoids from blood to the dermis and epidermis) may contribute only weakly to skin-color changes visible to an observer.

The psychophysical results in this study suggest that diet-linked skin-coloration is perceivably healthier and more attractive at *Δ*E of 1.37 and 1.55, respectively. This is in line with the results of Re et al.'s study [53] which recently investigated the level of oxygenated blood-color change associated with perceptible benefits in skin-color, finding that average color differences of *Δ*E 1.44 and 1.38 are required between faces for one to be reliably deemed more healthy and attractive, respectively. The regression analyses in the current study reveal that an additional fruit or vegetable portion per day results in a color change of *Δ*E 0.47 over all skin and 0.46 for facial skin over a six-week period. Taken together, these results suggest that perceptibly healthier and more attractive skin-coloration is achievable through relatively modest increases in fruit and vegetable consumption (of fewer than four portions per day). Lower thresholds may be found using alternate methodology. For example, reducing the interstimulus interval (100 ms) and removing the visual mask may decrease any attenuating effect caused by short-term memory. Thus, the thresholds reported here are conservative, and it is possible that even smaller dietary changes are able to produce perceptible benefits to skin-coloration. It must be noted, however, that our psychophysical study utilized a sample with a mean age of 18.9; it is possible that older participants will be less sensitive to skin-color changes as color acuity is reported to decline with age [59].

The current study provides further evidence that fruit and vegetable consumption affects skin-color. Diet-linked skin-color changes occurred over a relatively short time period and were attainable through relatively modest dietary changes; these conditions suggest potential utility as a dietary intervention tool. In order to verify whether wide-scale public health benefits could be reaped, it is important for further research to demonstrate that the effects extend to non-Caucasians and to populations with a greater range in initial diet.
